# Hydrogen Attenuates Chronic Intermittent Hypoxia-Induced Cardiac Hypertrophy by Regulating Iron Metabolism

**DOI:** 10.3390/cimb45120636

**Published:** 2023-12-16

**Authors:** Jixian Song, Qi Chen, Shan Xu, Yujing Gou, Yajing Guo, Cuiling Jia, Chenbing Zhao, Zhi Zhang, Boliang Li, Yashuo Zhao, Ensheng Ji

**Affiliations:** 1 Hebei Technology Innovation Center of TCM Combined Hydrogen Medicine, Hebei University of Chinese Medicine, Shijiazhuang 050200, China; smartsjx@163.com (J.S.); chenqi20202020@163.com (Q.C.); xushan20202020@163.com (S.X.); gyj.l@163.com (Y.G.); gyjayzw@163.com (Y.G.); zwfdc56@163.com (C.J.); zhaochenbing2022@163.com (C.Z.); 15680758958@163.com (Z.Z.); 13673117975@163.com (B.L.); 2 Department of Physiology, Institute of Basic Medicine, Hebei University of Chinese Medicine, Shijiazhuang 050200, China; 3 The First Affiliated Hospital, Hebei University of Chinese Medicine, Shijiazhuang 050013, China

**Keywords:** obstructive sleep apnea, hydrogen, chronic intermittent hypoxia, cardiac hypertrophy, mitochondrial dysfunction, ferroportin 1, hepcidin

## Abstract

The present study aimed to investigate the impact of hydrogen (H_2_) on chronic intermittent hypoxia (CIH)-induced cardiac hypertrophy in mice by modulating iron metabolism. C57BL/6N mice were randomly allocated into four groups: control (Con), CIH, CIH + H_2_, and H_2_. The mice were exposed to CIH (21–5% FiO_2_, 3 min/cycle, 8 h/d), and received inhalation of a hydrogen–oxygen mixture (2 h/d) for 5 weeks. Cardiac and mitochondrial function, levels of reactive oxygen species (ROS), and iron levels were evaluated. The H9C2 cell line was subjected to intermittent hypoxia (IH) and treated with H_2_. Firstly, we found H_2_ had a notable impact on cardiac hypertrophy, ameliorated pathological alterations and mitochondrial morphology induced by CIH (*p* < 0.05). Secondly, H_2_ exhibited a suppressive effect on oxidative injury by decreasing levels of inducible nitric oxide synthase (i-NOS) (*p* < 0.05) and 4-hydroxynonenal (4-HNE) (*p* < 0.01). Thirdly, H_2_ demonstrated a significant reduction in iron levels within myocardial cells through the upregulation of ferroportin 1 (FPN1) proteins (*p* < 0.01) and the downregulation of transferrin receptor 1 (TfR1), divalent metal transporter 1 with iron-responsive element (DMT1(+ire)), and ferritin light chain (FTL) mRNA or proteins (*p* < 0.05). Simultaneously, H_2_ exhibited the ability to decrease the levels of Fe^2+^ and ROS in H9C2 cells exposed to IH (*p* < 0.05). Moreover, H_2_ mediated the expression of hepcidin, hypoxia-inducible factor-1α (HIF-1α) (*p* < 0.01), and iron regulatory proteins (IRPs), which might be involved in the regulation of iron-related transporter proteins. These results suggested that H_2_ may be beneficial in preventing cardiac hypertrophy, a condition associated with reduced iron toxicity.

## 1. Introduction

Obstructive sleep apnea (OSA) is a highly prevalent sleep disorder that has been associated with various cardiovascular diseases, such as hypertension, coronary artery disease, and cardiac hypertrophy [1]. Chronic intermittent hypoxia (CIH), a significant pathological change in OSA, induces the generation of reactive oxygen species (ROS), leading to oxidative damage or apoptosis [2]. The ROS exerts its influence on the cardiac membrane, leading to the degradation of its structure and functionality, ultimately resulting in the autolysis of cardiomyocytes [3]. Moreover, recurrent episodes of apnea elevate intrathoracic pressure, increasing the left ventricular afterload and ultimately inducing cardiac hypertrophy [4].

Iron is an essential micronutrient in the human body, involved in critical physiological processes, such as electron transport, cellular respiration, and DNA synthesis [5]. However, it is important to maintain appropriate levels of iron, as excessive accumulation could accelerate the generation of ROS through the Fenton reaction, ultimately exacerbating oxidative damage [6]. Iron homeostasis is crucial for the proper functioning of various mammalian cell types, with particular emphasis on cardiovascular well-being; research has demonstrated that iron overload could give rise to left ventricular hypertrophy and ferroptosis, ultimately leading to cardiac impairment in mice lacking H ferritin subunit [7,8]. The positive impact of administering desferrioxamine (DFO), an iron-chelating agent, on cardiac hypertrophy has been observed [9,10,11], indicating that iron deposition may contribute to the development of cardiac hypertrophy. 

Our previousstudy has demonstrated that inhaling hydrogen (H_2_) under CIH could effectively attenuate iron overload in renal tubular epithelial cells and renal injury [12]. H_2_ demonstrates a multitude of beneficial effects, including antioxidant, anti-inflammatory, anti-apoptotic properties, and can efficiently disperse throughout the entirety of the body, owing to its small size and light weight [13]. Moreover, H_2_ exhibits a remarkable absence or minimal incidence of unfavorable reactions, as it does not hinder metabolic processes, REDOX reactions, or the generation of ROS involved in cellular signaling events [14]. Clinical examination revealed that exposure to 2.4% H_2_ gas for 72 h did not induce any alterations in physiological indices [15]. Currently, studies have shown that modalities including hydrogen-enriched saline and hydrogen inhalation can improve ischemia-reperfusion injury and left ventricular hypertrophy in rats [16,17].

However, regarding the role of H_2_, current studies mostly focus on oxidative stress, inflammation, and other aspects, and there are few studies on the relationship between H_2_ and iron metabolism under hypoxia. In this study, we aimed to examine the impact of H_2_ on cardiac iron metabolism by utilizing a rodent model of CIH. On the one hand, it bridges the research gap between H_2_ and iron metabolism, and on the other hand, it aims to provide a theoretical basis for therapeutic intervention in cardiovascular injury in patients with OSA.

## 2. Materials and Methods

### 2.1. Animals

Male *C57BL/6N* mice (SPF grade, 6 weeks) were purchased from Beijing Vital River Laboratory Animal Technology Co., Ltd., Beijing, China. Subsequently, all mice were transferred to the Experimental Animal Center, where they were maintained under SPF conditions and given enough water and food (HUANYU BIO, HD8013, Moisture ≤ 100, Crude Protein ≥ 200, Crude Fat ≥ 40, Crude Fiber ≤ 50, Crude Ash ≤ 80), subjected to a controlled light–dark cycle of 12 h each while ensuring regulated temperature conditions. The animal experimental procedures adhered to the guidelines set forth by the National Institutes of Health Guide for the Care and Use of Laboratory Animals and were duly approved by the Animal Care and Use Committee of Medical Ethics of Hebei University of Chinese Medicine (Animal Ethics Number, DWLL2021097).

A total of 40 *C57BL/6N* mice were randomly divided into four groups: the normal oxygen control group (Con), the chronic intermittent hypoxia (CIH) group, the CIH+H_2_ group, and the H_2_ group. The CIH and CIH+H_2_ mice were exposed to controlled hypoxia using an OxyCycler chamber (BioSpherix Ltd., Parish, NY 13131, USA) to simulate an OSA mouse model. During the first 1.5 min of each session, the chamber was filled with 100% N_2_ to decrease the percentage of O_2_ in breathing air from 21% to 5%. In the last 1.5 min, O_2_ was gradually reintroduced to restore the O_2_ level to 21%. Each hypoxic–reoxygenation session had a duration of 3 min and was conducted repeatedly for 8 h (from 9:00 to 17:00) per day for 35 days. The mice in the Con group were placed in the same chamber, filled with normal air (21% O_2_). The mice in the CIH+H_2_ and H_2_ groups were transferred to a transparent closed box and administered H_2_–O_2_ mixture gas from 17:00 to 19:00 each day throughout the 35 days. The H_2_–O_2_ mixture gas was generated through water electrolyzation using a hydrogen–oxygen nebulizer (AMS-H-01, Shanghai Asclepius Meditec Co., Ltd., Shanghai, China) [18] and comprised 66% hydrogen and 33% oxygen. In addition, the mice in the Con and CIH groups received normal air in the same box.

### 2.2. Echocardiography

Echocardiographic analysis was conducted using a high-resolution ultrasound imaging system (Vevo 2100, VisualSonic Inc., Toronto, ON, Canada) equipped with an MS-250 probe to assess cardiac function in mice. Before the analysis, the mice were anaesthetized with a 2.5% isoflurane mixture containing 5% CO_2_ and 95% O_2_. Subsequently, depilatory cream was applied to remove hair from the chest area, and an ample amount of ultrasonic coupling agent was applied to the chest wall. The ejection fraction (EF), fractional shortening (FS), left ventricular end-systolic diameter (LVESD), left ventricular end-diastolic dimension (LVEDD), and left ventricular posterior wall depth (LVPWD) were quantified through the utilization of M-mode recording of the short-axis view.

### 2.3. Histopathological Examinations

The cardiac paraffin sections underwent deparaffinization and rehydration, followed by treatment with an EDTA antigen repair solution for antigen repair. Subsequently, the sections were washed and incubated with FITC-labeled WGA solution (a cell surface stain, 1:200, I3300, Solarbio, Beijing, China) for 60 min at 37 °C, while being shielded from light. Afterwards, the sections were exposed to DAPI at room temperature for 10 min. Ultimately, the sections were sealed using an anti-fade sealer and examined and captured using a fluorescence microscope.

### 2.4. Mitochondrial Membrane Potential Measurement

Firstly, mitochondria were isolated following the protocols outlined in the Mitochondrial Extraction Kit (MP-007, Invent Biotechnologies, Aurora, CO, USA). The myocardial tissue from each experimental group was weighed and prepared as a 10% homogenate. The resulting homogenate was subjected to centrifugation at 2000 rpm for 10 min using a low-temperature high-speed centrifuge, and the precipitate was discarded. The supernatant was then subjected to further centrifugation at 10,000 rpm for 15 min, resulting in the isolation of the mitochondrial sediment. Subsequently, the JC-1 mitochondrial membrane potential detection kit (G1515-100T, Servicebio, Wuhan, China) was employed according to its instructions to carry out the JC-1 staining process. Finally, the fluorescence value was measured by a multifunctional microplate reader microplate in Em 425–520 nm, Ex 485 nm (Varioskan LUX, Thermo Fisher Scientific, former Savant, MA, USA).

### 2.5. Immunohistochemistry

Paraffin sections were dewaxed, rehydrated, and then incubated with 3% H_2_O_2_ to remove endogenous peroxidase. The sections received antigen retrieval performed via high temperature using citrate antigen repair buffer (10 mM, pH 6.0). Then the sections were blocked with 10% goat serum for 1 h at 37 °C. The cardiac sections were incubated with primary antibodies: nuclear factor erythroid 2-related factor 2 (Nrf2, 66504-1-Ig, Proteintech, Wuhan, China), ferroportin 1 (FPN1, MTP11-A, Alpha Diagnostic International, San Antonio, TX 78249, USA), hepcidin (DF6492, Affinity Biosciences, Cincinnati, OH, USA), hypoxia-inducible factor 1-alpha (HIF1-α, PB9253, Boster, Wuhan, China) overnight at 4 °C. On the second day, sections were incubated with HRP-conjugated second antibody for 1 h at 37 °C. Then, sections were enhanced and stained with a diaminobenzidine (DAB) kit. After being sealed with neutral balsam, images were taken, and the average density was stated by IPP 6.0 software.

### 2.6. Transmission Electron Microscope

The ultrastructure of mitochondria was observed by transmission electron microscope (TEM). After the mice were anaesthetized, left ventricular tissue (1 mm^3^) was quickly taken and immersed in an electron microscopy fixation solution for 2–4 h. Then the tissue was osmotically embedded, polymerized, and cut into ultrathin sections of 60–80 nm. After staining with uranyl acetate and lead nitrate, the images were observed under a transmission electron microscope and analyzed under an electron microscope (HT7800, HITACHI, Tokyo, Japan).

### 2.7. Perls’ Staining

The paraffin sections underwent a series of procedures including dewaxing, rehydration, and rinsing with 0.01 M PBS. Subsequently, the sections were subjected to blocking with 3% H_2_O_2_, a blocker of endogenous catalase, for 30 min. Following this, the sections were washed with PBS and immersed in a solution of 1% fresh Prussian blue dye, which is a mixture of potassium ferrocyanide and hydrochloric acid. The sections were then incubated in darkness at a temperature of 37 °C for a period of 12 h. After being washed with PBS, DAB was employed to enhance color development. Finally, the sections were dehydrated using a gradient and sealed with film. The resulting images were visualized, and the mean density of iron content was calculated using IPP 6.0 software.

### 2.8. Determination of Total Iron

The total iron content was measured according to its specification (A039-2-1, Nanjing Jiancheng Bioengineering Institute, Nanjing, China). Approximately 50 mg of cardiac tissue was weighed and subsequently mixed with nine times the volume of saline. Mechanical homogenization was performed under an ice water bath, followed by centrifugation. The resulting supernatant was combined with three times the volume of iron colorant and thoroughly mixed. The mixture was then incubated at a temperature of 95 °C for 5 min. After cooling and centrifugation, the supernatant was subjected to absorbance measurement at 520 nm. The total iron content was calculated by formula according to the specification.

### 2.9. Q-PCR

The extraction of total RNA from heart tissue was carried out using the RNA extraction kit (DP419, Tiangen Biotech, Beijing, China) according to the provided instructions. Subsequently, the extracted total RNA (1 μg) was subjected to reverse transcription into cDNA using the PrimeScript TM RT regent Kit with gDNA Eraser (RR047A, Takara, Beijing, China). The quantification of PCR amplification was performed using the SYBR-Green PCR Master Mix kit (RR820A, Takara, Beijing, China). The expression levels of atrial natriuretic peptide type A *(Nppa)*, natriuretic peptide type B *(Nppb)*, myosin heavy chain 7 *(MYH7)*, Mitochondrial Fission 1 *(Fis-1)*, Mitochondrial dynamin-like GTPase *(Opa-1)*, Dynamin-related protein 1 *(Drp-1)*, Divalent metal transporter 1(+ire) *(DMT1(+ire))*, Divalent metal transporter 1*(−ire) (DMT1(−ire))*, *FPN1*, and *hepcidin* mRNA were determined using quantitative PCR (Q-PCR). The primer sequences used for Q-PCR are provided in Table 1. The relative gene expression was calculated by the 2^−ΔΔCt^ formula.

### 2.10. Western Blot

Initially, the pre-cooled RIPA lysate was added to the heart tissue, followed by centrifugation and the preparation of a homogenate with a concentration of 10% g/V. Subsequently, the protein concentration was assessed using the BCA method. The entirety of proteins was subsequently separated through SDS-PAGE and then transferred onto polyvinylidene fluoride (PVDF) membranes. Following the application of a 5% skim milk powder block, the blots were incubated with primary antibodies Fis-1 (505821, Zen-bioscience, Chengdu, China), Opa-1 (Zen-bioscience, 382025, Chengdu, China), Drp-1 (Zen-bioscience, 340336, Chengdu, China), 4-hydroxynonenal (4-HNE, ARG23717, Arigo Biolaboratories, Hsinchu, Taiwan), Inducible nitric-oxide synthase (i-NOS, GB11119, Servicebio, Wuhan, China).

Nrf2, Kelch-like Ech-related protein 1 (Keap1, bs-4900R, BIOSS, Beijing, China), Heme Oxygenase 1 (HO-1, Abclonal, Wuhan, China, A1346), FPN1, Transferrin Receptor Protein 1 (TfR1, abs120325, Absin, Shanghai, China), Ferritin Heavy chain (FTH, ab183781, Abcam, Cambridge, MA, USA), Ferritin Light chain (FTL, ab218400, Abcam, Cambridge, MA, USA), Mitochondrial Ferritin (MtFt, ab66111, Abcam, Cambridge, MA, USA), HIF-1α, Iron Regulatory Protein 1 (IRP-1, 20272S, Cell Signaling Technology, Danvers, MA, USA), Iron Regulatory Protein 2 (IRP-2, 37135S, Cell Signaling Technology, Danvers, MA, USA), F-box and leucine-rich repeat protein 5 (FBXL5, GTX32599, GeneTex, San Antonio, TX, USA), β-actin (GB15001, Servicebio, Wuhan, China), α-Tubulin (GTX628802, GeneTex, San Antonio, TX, USA), and GAPDH (Servicebio, GB15002, Wuhan, China) overnight at 4 °C. The next day, secondary antibodies were prepared according to the source of primary antibodies and incubated for 1.5 h at room temperature. Finally, immunoreactive proteins were imaged by the ECL method. Image J software 6.1 was used to analyze the average grey value of the target band, and SPSS 22.0 software was used for statistical analysis.

### 2.11. Cell Culture and Associated Assay

#### 2.11.1. Preparation of Hydrogen-Rich Media

The hydrogen-rich media were prepared by dissolving hydrogen in the media for a duration of 6 h under high-pressure conditions (0.4 MPa) to achieve saturation. The hydrogen content was determined to be 0.8 ppm. Subsequently, the resulting hydrogen-rich media were packed, stored, disinfected through radiation disinfection, and deemed ready for utilization. 

#### 2.11.2. Cell Culture

H9C2 cells were cultured in DMEM medium supplemented with 10% fetal bovine serum, penicillin, and streptomycin at a temperature of 37 °C with a CO_2_ concentration of 5%. In the control group, cells were cultivated under normal oxygen conditions. An H9C2 cell model of intermittent hypoxia (IH) was established for a duration of 24 h, and each cycle consisting of 0.1% O_2_ for 3 min followed by 21% O_2_ for 7 min. After IH treatment, the cells were exposed to hydrogen-rich media for a duration of 1 h.

#### 2.11.3. Cell Viability Assay

Cell viability was evaluated by Cell Counting Kit-8 (CCK-8, 40203ES76, Yesen, Shanghai, China). H9C2 cells were seeded at a density of 1 × 10^4^ cells per well in 96-well culture plates. Following treatment with or without IH and H_2_, the CCK-8 reagent was added to the plates and incubated at 37 °C for 1 h. Subsequently, the absorbance was quantified at 450 nm utilizing a multifunctional microplate reader (Varioskan LUX, Thermo Fisher Scientific, Shanghai, China).

#### 2.11.4. FerroOrange Staining

The FerroOrange probe (MX4580, Maokangbio, Shanghai, China) was used to examine the presence of Fe^2+^ in H9C2 cells. The cells were seeded onto 6 cm cell culture dishes at a density of 2 × 10^5^. Before staining, the cells were prepared by removing the supernatant and washing them three times with PBS. Subsequently, a staining solution containing 1 uM of FerroOrange was added to the cells, which were then incubated for 30 min at 37 °C and 5% CO_2_ in an incubator. Subsequently, the cells were subjected to observation and documentation employing a fluorescence microscope, characterized by an emission wavelength of 532 nm.

#### 2.11.5. ROS Levels

Briefly, H9C2 cells were seeded at a density of 1 × 10^4^ cells per well in 96-well culture plates one day before the treatment, and then treated with or without IH for 24 h, followed by treatment with H_2_ for 1 h. Dilute DCFH-DA (Servicebio, G1706, Wuhan, China) with serum-free culture medium according to 1:1000 to give a final concentration of 10 µM. The cell culture solution was removed and the appropriate volume of diluted DCFH-DA was added. Cells were then incubated in a humidified incubator at 37 °C and 5% CO_2_ for 20 min. Subsequently, the cells were washed three times with PBS, and the fluorescence intensity was quantified by a multifunctional microplate reader microplate (Ex/Em = 488 nm/525 nm).

#### 2.11.6. Immunofluorescence Double Staining

The cells subjected to treatment were harvested upon reaching 95–100% confluency on coverslips, followed by three rinses with PBS. Subsequently, the cells were fixed with paraformaldehyde and permeabilized with Triton X-100 (T8200, Solarbio, Beijing, China). Following this, the cells were incubated at room temperature, exposed to primary and secondary antibodies (FPN1 and hepcidin), and sealed with an anti-fluorescence quenching sealer containing DAPI. Ultimately, the cells were examined and captured using a fluorescence microscope.

### 2.12. Statistical Analysis

All the experimental data were analyzed by SPSS 23.0 statistical software. The experimental data were expressed as mean ± SEM, and statistically analyzed by one-way ANOVA followed by the LSD *post hoc test*. The significance level was considered as *p* < 0.05. Statistical maps were drawn using Prism 8.0 software.

## 3. Results

### 3.1. Results

#### 3.1.1. Hydrogen Improved Cardiac Hypertrophy and Histological Changes Induced by CIH

**Figure 1:**
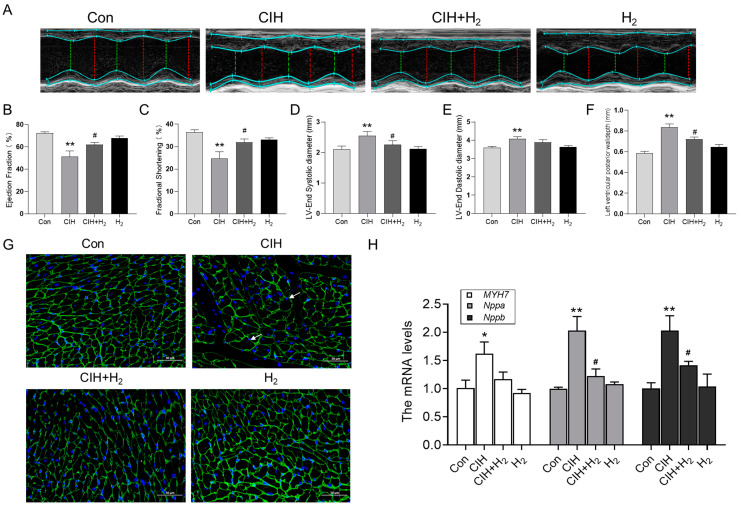
The cardiac hypertrophy and dysfunction induced by CIH exposure in mice. (**A**) M-model echocardiography in mice (*n* = 6). (**B**) The ejection fraction of the left ventricle (*n* = 6). (**C**) The fractional shortening (*n* = 6). (**D**) The left ventricular end-diastolic diameter (*n* = 6). (**E**) The left ventricular end-systolic diameter (*n* = 6). (**F**) The left ventricular posterior wall depth (*n* = 6). (**G**) The WGA immunofluorescence stain from Con, CIH, CIH+H_2_, and H_2_ groups (scale bar = 50 μm, *n* = 3). (**H**) The *MYH7*, *Nppa*, and *Nppb* mRNA levels in heart tissue. The data are presented as the means ± SEM. * *p* < 0.05, ** *p* < 0.01 vs. Con group. ^#^ *p* < 0.05 vs. CIH group.

As depicted in Figure 1, the representative M-mode parasternal short-axis view was utilized to assess the systolic and diastolic function. The left ventricular ejection fraction (EF) and short-axis fractional shortening (FS) of CIH mice exhibited a decrease (Figure 1B,C), while the average values of left ventricular end-systolic diameter (LVESD) and left ventricular end-diastolic diameter (LVEDD) were elevated compared to normal mice (Figure 1D,E). The characteristic index of myocardial hypertrophy left ventricular posterior wall thickness at end-diastole (LVPWD) was notably increased in the CIH group (Figure 1F). In summary, these indicators demonstrated significant improvement following H_2_ treatment, as evidenced by the CIH+H_2_ group (Figure 1). The WGA immunofluorescence stain revealed that the cross-sectional area of cardiomyocytes in the CIH group was significantly larger compared to the Con group. However, the administration of H_2_ appeared to alleviate this trend, as depicted in Figure 1G. Additionally, the Q-PCR results demonstrated a significant increase in *MYH7*, *Nppa*, and *Nppb* mRNA levels in the CIH group, which were subsequently reduced in the CIH+H_2_ group, as shown in Figure 1H. Taken together, these findings suggested that exposure to CIH led to myocardial hypertrophy, whereas the administration of H_2_ effectively mitigated the associated pathological damage.

#### 3.1.2. Hydrogen Mitigated the Mitochondrial Dysfunction Induced by CIH

**Figure 2:**
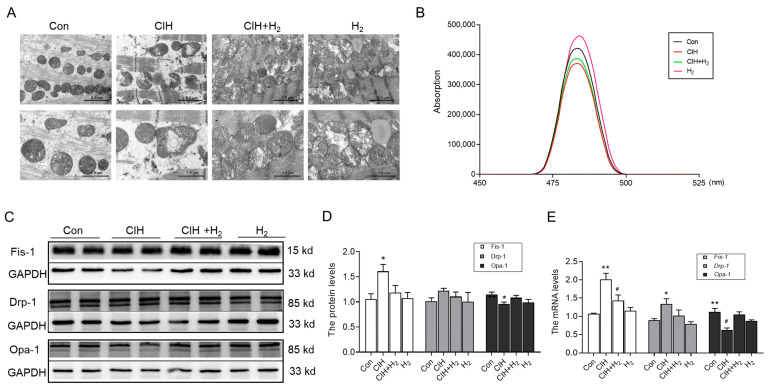
The mitochondrial damage in the heart of CIH mice. (**A**) The TEM images of mitochondria in the heart (scale bar = 2 or 1 μm, *n* = 3). (**B**) The mitochondrial membrane potential (*n* = 6). (**C**,**D**) The expression and statistics of Fis-1, Drp-1, and Opa-1 protein levels (*n* = 6). (**E**) The *Fis-1*, *Drp-1*, and *Opa-1* mRNA levels in heart tissue (*n* = 3). The data are presented as the means ± SEM. * *p* < 0.05, ** *p* < 0.01 vs. Con group. ^#^ *p* < 0.05 vs. CIH group.

The heart possesses a substantial abundance of mitochondria, which serve as a source of energy, but they are also susceptible to damage. The TEM images revealed noticeable impairment and structural incompleteness in the mitochondrial spines of the myocardium in CIH mice, as compared to normal mice (Figure 2A). Conversely, the mice in the CIH+H_2_ group exhibited a relatively intact mitochondrial structure in the heart, in comparison to CIH mice (Figure 2A). The JC-1 probe was employed to assess the mitochondrial membrane potential. As depicted in Figure 2B, the mitochondrial membrane potential level in the CIH group exhibited a significant decrease, which was ameliorated by treatment with H_2_. The Western blot analysis revealed an increase in Fis-1 protein expression, a non-significant increase in Drp-1 protein expression, and a decrease in Opa-1 protein expression in the CIH group (Figure 2C,D). Following treatment with the H_2_, these protein levels demonstrated a corresponding improvement. The mRNA results for *Fis-1*, *Drp-1*, and *Opa-1* exhibited a similar trend to the protein levels (Figure 2E). This evidence supports the conclusion that H_2_ has a significant impact on reducing CIH-induced mitochondrial damage.

#### 3.1.3. Hydrogen Efficiently Inhibited Oxidative Stress in Cardiac Tissue Induced by CIH

**Figure 3:**
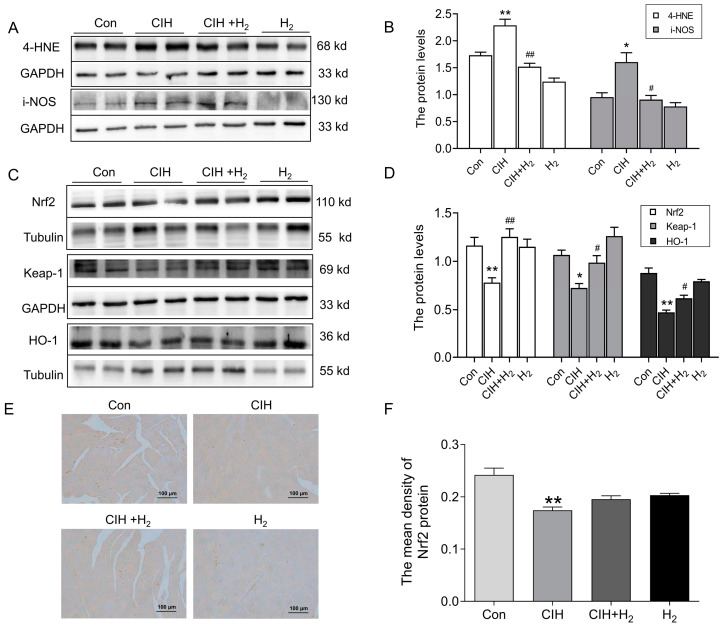
The oxidative stress level in the cardiac tissue subjected to CIH. (**A**,**B**) The expression and statistics of 4-HNE and i-NOS protein levels (*n* = 4–6). (**C**,**D**) The expression and statistics of Nrf2, Keap-1, and HO-1 protein levels (*n* = 6). (**E**,**F**) The immunohistochemical staining and statistics of Nrf2 protein (scale bar = 100 μm, *n* = 3). The results are presented as the mean ± SEM. * *p* < 0.05, ** *p* < 0.01 vs. Con group. ^#^ *p* < 0.05, ^##^ *p* < 0.01 vs. CIH group.

Mitochondrial abnormalities frequently contribute to the occurrence of oxidative stress and subsequent damage. The extent of oxidative stress was assessed through the estimation of antioxidant capacity and lipid peroxidation. Notably, the heart tissue of CIH mice exhibited a significant increase in 4-HNE and i-NOS protein levels, as depicted in Figure 3A,B, signifying the reception of oxidative damage. Simultaneously, the Nrf2, Keap1, and HO-1 protein levels exhibited a decrease in the cardiac tissue of the CIH group in comparison to the Con group, as shown in Figure 3C,D. This decrease suggested a compromised antioxidant capacity of H_2_. Additionally, the immunohistochemistry (IHC) analysis revealed a reduction in Nrf2 protein levels following exposure to CIH. However, treatment with H_2_ demonstrated the ability to reverse the protein levels, as shown in Figure 3E,F. Consequently, these findings indicated that H_2_ had the potential to enhance antioxidant capacity and mitigate peroxide-induced damage. 

#### 3.1.4. Hydrogen Decreased Iron Deposits in the Cardiac Tissue of CIH Mice by Upregulating FPN1

**Figure 4:**
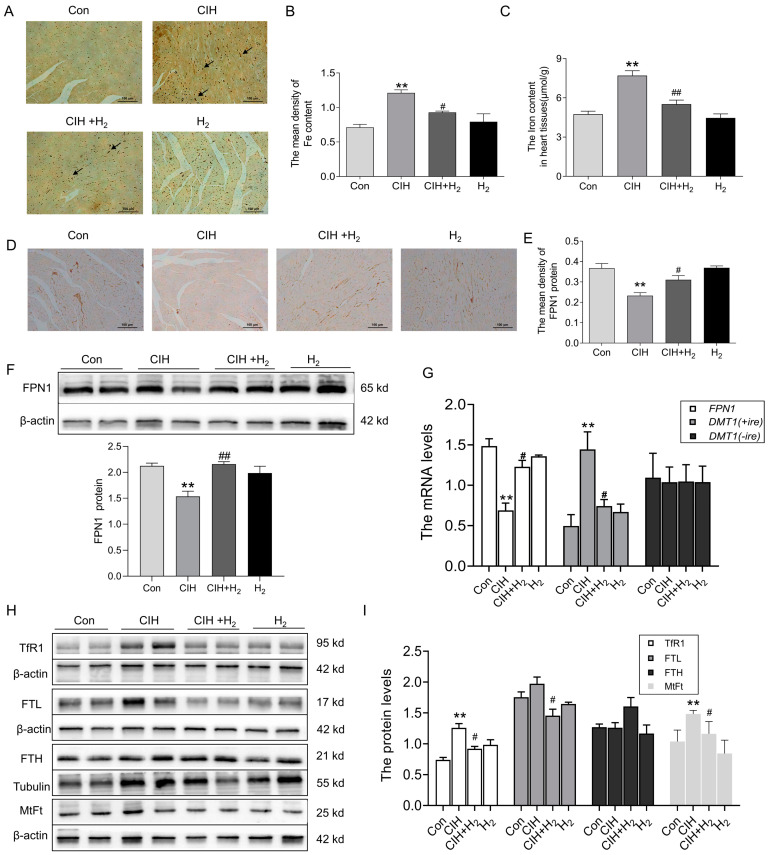
The iron content and iron-related transport proteins in the cardiac tissue during CIH. (**A**) The Perls’ staining of heart tissue (scale bar = 100 μm, *n* = 3). (**B**) The mean density of Fe content as shown in panel A. (**C**) The total iron content in the cardiac tissue (*n* = 5). (**D**,**E**) The immunohistochemical staining of FPN1 protein (scale bar = 100 μm, *n* = 3). (**F**) The expression and statistics of FPN1 protein levels were measured by Western blot (*n* = 6). (**G**) The FPN1, DMT1(+ire), DMT1(-ire) mRNA levels in heart tissue (*n* = 6). (**H**,**I**) The expression and statistics of TfR1, FTL, FTH, and MtFt protein levels (*n* = 6). The results are presented as the mean ± SEM. ** *p* < 0.01 vs. Con group. ^#^ *p* < 0.05, ^##^ *p*< 0.01 vs. CIH group.

Excessive iron levels have been found to stimulate the production of ROS and exacerbate oxidative damage. To investigate this further, we analyzed iron levels in heart tissue following exposure to CIH. As shown in Figure 4A,B, Perls’ staining revealed a significant increase in ferric iron content within the cardiomyocytes of CIH mice. At the same time, the total iron content in cardiac tissue was elevated after exposure to CIH, which was decreased after administration of H_2_ (Figure 4C)_._ Additionally, IHC staining (Figure 4D,E) and Western blot analysis (Figure 4F) revealed a decrease in the expression of the FPN1 protein after CIH treatment, with levels returning to baseline following treatment with H_2_. Consistent with these results, the mRNA levels of *FPN1* exhibited a similar trend (Figure 4G). The mRNA level of *DMT1(+ire)* exhibited a significant increase, whereas the mRNA level of *DMT1(-ire)* showed minimal change (Figure 4G). The CIH group demonstrated higher levels of TfR1, the primary protein responsible for iron uptake, compared to the Con group (Figure 4H). Additionally, the CIH group exhibited elevated levels of iron storage proteins, FTL and MtFt, while the FTH protein level displayed a non-significant increase (Figure 4H,I). Administration of H_2_ resulted in an increase in FPN1 level and a decrease in DMT1(+ire), TfR1, FTL, and MtFt levels, thereby maintaining iron metabolic balance in heart tissue during exposure to CIH.

#### 3.1.5. Hydrogen Inhibited Iron Deposition by Regulating Hepcidin

**Figure 5:**
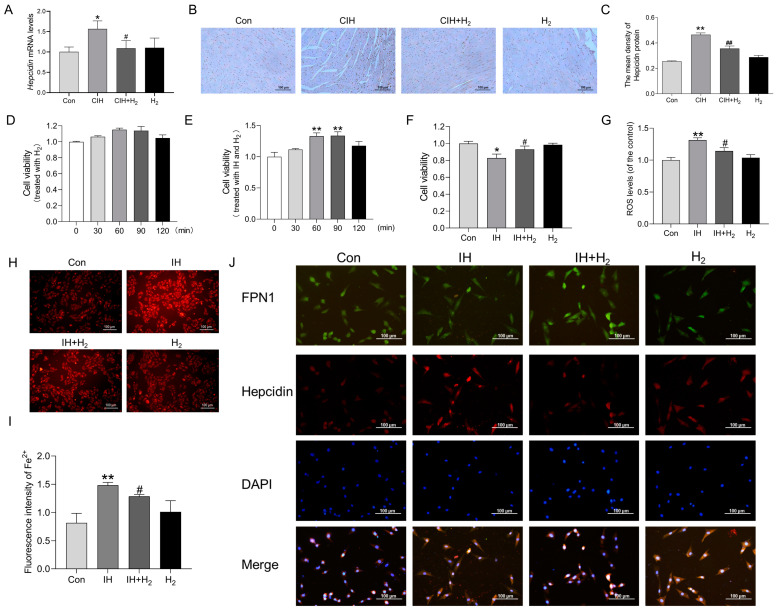
The Hepcidin level in the cardiac tissue during CIH and the effects on H9C2 cells after IH exposure. (**A**) The *Hepcidin* mRNA levels in heart tissue (*n* = 3). (**B**,**C**) The immunohistochemical staining of Hepcidin protein (scale bar = 100 μm, *n* = 3). (**D**) The cell viability of H9C2 cells treated with hydrogen for 0, 30, 60, 90, and 120 min (*n* = 6). (**E**) The cell viability of H9C2 cells treated with IH and hydrogen for 0, 30, 60, 90, and 120 min (*n* = 5). (**F**) The cell viability of H9C2 cells treated with 60 min (*n* = 6). (**G**) The ROS level induced by IH (*n* = 6). (**H**,**I**) The fluorescence intensity of Fe^2+^ (scale bar = 100 μm, *n* = 3). (**J**) The immunofluorescence double-label staining of FPN1 and Hepcidin (scale bar = 100 μm, *n* = 3). The results are presented as the mean ± SEM. * *p* < 0.05, ** *p* < 0.01 vs. Con group. ^#^ *p* < 0.05, ^##^ *p* < 0.01 vs. IH group.

We conducted further investigation into the potential mechanism by which H_2_ may upregulate the expression of FPN1. Firstly, we evaluated the expression of hepcidin mRNA and protein levels. Q-PCR analysis revealed an increase in *hepcidin* mRNA levels following exposure to CIH (Figure 5A); at the same time, IHC imaging demonstrated a noticeable increase in hepcidin protein levels in the CIH group compared to the Con group (Figure 5B,C).

To further investigate the potential of H_2_ in mitigating myocardial iron deposition and ROS injury induced by CIH through modulation of FPN1, we conducted an *in vitro* study using cultured H9C2 cardiomyocytes. As shown in Figure 5D, H_2_ treatment alone did not affect cell viability. An IH model was established, and our findings indicated that elevated cell viability significantly increased following IH exposure when treated with H_2_ for 60 min (Figure 5E,F). Additionally, the results from the DCFH-DA probe revealed that the increased ROS level induced by IH declined when treated with H_2_ (Figure 5G). The FerroOrange probe demonstrated a significant reduction in the labile iron pool (LIP) after 60 min of H_2_ treatment in the IH model (Figure 5H,I). Finally, immunofluorescence double-label staining indicated a significant increase in hepcidin protein expression and a decrease in FPN1 protein expression in H9C2 cells following exposure to IH (Figure 5J). Conversely, the H_2_-treated group exhibited contrasting results (Figure 5J). These observations align with our in vivo results, suggesting that H_2_ treatment could effectively regulate hepcidin-FPN1 and mitigate iron deposition.

#### 3.1.6. Hydrogen Upregulated HIF-1α Expression

**Figure 6:**
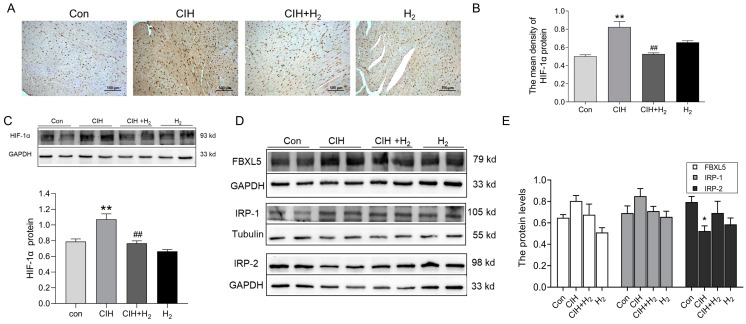
The HIF-1α and related proteins in the cardiac tissue during CIH. (**A**,**B**) The immunohistochemical staining of HIF-1α protein (scale bar = 100 μm, *n* = 3). (**C**) The expression and statistics of HIF-1α protein levels (*n* = 6). (**D**,**E**) The expression and statistics of FBXL5, IRP-1, and IRP-2 protein levels (*n* = 6). The results are presented as the mean ± SEM. * *p* < 0.05, ** *p* < 0.01 vs. Con group. ^##^ *p* < 0.01 vs. CIH group.

We assessed the expression of hypoxia signaling and iron regulatory proteins in cardiac tissue. IHC analysis indicated an increase in HIF-1α protein levels in the heart tissue of CIH mice, which was subsequently followed by a decline after H_2_ treatment (Figure 6A,B). Similarly, the Western blot showed similar results as shown in Figure 6A,B (Figure 6C). In addition, we examined the expression of iron-regulatory proteins. As shown in Western blot results, FBXL5, and IRP-1 protein levels were increased and IRP-2 protein levels declined in the CIH group (Figure 6D,E). Of these, the most significant changes were in IRP-2. However, the expression patterns of these proteins could be slightly reversed by the administration of H_2_. These findings suggest the potential involvement of HIF-1α and related genes in the regulation of FPN1.

## 4. Discussion

In this study, a CIH mouse model was developed to simulate OSA, and it was determined that iron deposition and oxidative stress damage were associated with CIH-induced myocardial hypertrophy. The inhalation of H_2_ was found to prevent iron deposition by upregulating FPN1 expression. Additionally, hepcidin and IRPs might all be involved in the regulation of FPN1. Consequently, the H_2_ may serve as a secure therapeutic approach for conditions characterized by iron deposits.

There is empirical evidence suggesting that OSA plays a role in the progression of cardiovascular dysfunction. CIH is recognized as the primary pathological characteristic of OSA, as repeated episodes of oxygen deprivation could result in left ventricular hypertrophy and subsequent cardiac dysfunction [19]. To replicate the intermittent hypoxic episodes experienced by OSA patients, we have established a CIH-exposed model. Lee et al. have demonstrated that CIH could induce an increase in ventricular wall thickness and significant alterations in myocardial structure in rats [20]. Consistently, our findings indicated that exposure to CIH resulted in elevated LVES, LVEDD, and LVPWD in mice. Additionally, CIH exposure led to significant enlargement of cardiomyocytes, as evidenced by increased cross-sectional area observed through WGA staining. Moreover, the expression levels of *Nppa* and *Nppb*, which played crucial roles in combating ventricular remodeling and maintaining cardiac structure [21], were found to be significantly elevated after CIH exposure (Figure 1H). Furthermore, the *MYH7* gene, specifically associated with hypertrophic cardiomyopathy, also exhibited a significant increase following CIH exposure [22]. All the results point to CIH exposure being able to cause cardiac hypertrophy.

**Figure 7:**
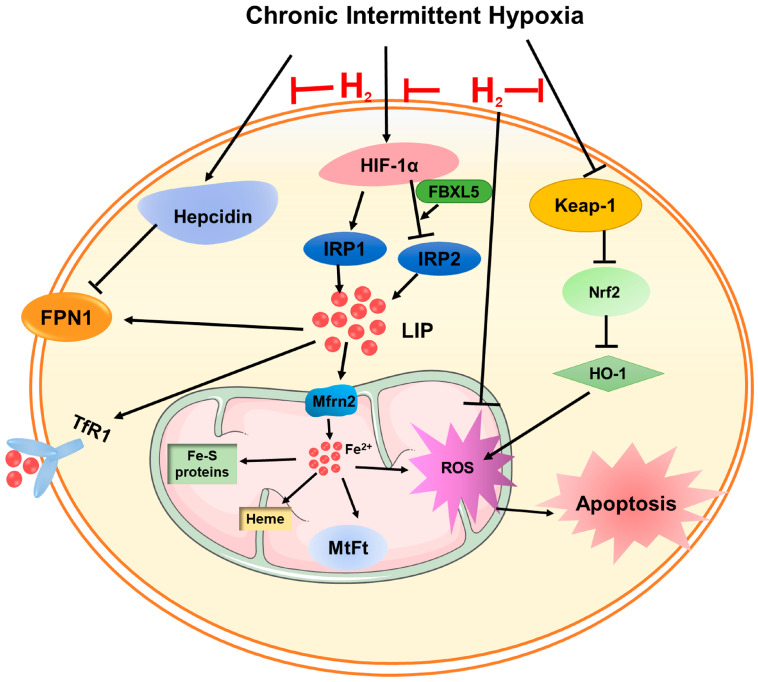
A schematic representation of the proposed cardioprotective mechanism of hydrogen after CIH exposure. On the one hand, hydrogen attenuated ROS levels through the Keap-1/Nrf2 signaling pathway. On the other hand, hydrogen could inhibit iron overload-induced ROS generation and oxidative damage by adjusting hepcidin-FPN1, and HIF-1α-related IRPs’ signaling pathways.

H_2_ possesses robust antioxidant, anti-inflammatory, and anti-apoptotic properties, enabling it to permeate most cell membranes and influence the progression of cardiovascular diseases [23,24,25]. Studies have shown that H_2_ could combat mitochondrial degeneration, remodeling of the left ventricle caused by intermittent hypoxia in sleep apnea syndrome, and improve heart function [26]. Studies have also confirmed that H_2_ had a protective effect on the cardiovascular system, inducing smooth muscle relaxation, inhibiting the development of atherosclerosis, and that regular consumption of hydrogen-rich water could protect against inflammatory heart disease damage [27,28]. The findings of this study indicated that CIH exposure was capable of inducing cardiac hypertrophy, while H_2_ had the potential to mitigate the resulting damage (Figure 7).

Mitochondria, being a crucial organelle within the cell, exerts a significant influence on the progression of myocardial hypertrophy. Some studies have demonstrated that the disruption of mitochondrial structure and function, leading to energy metabolism disorders, could precipitate the onset of myocardial hypertrophy [29]. The emergence of cardiac hypertrophy is intricately linked to mitochondrial oxidative stress, which serves as the primary origin of ROS. Numerous investigations have substantiated that the inhibition of ROS generation could markedly ameliorate cardiac hypertrophy [3,30]. The findings of our experiment revealed that the mitochondrial structure exhibited evident damage, including membrane structure destruction and shrinkage, accompanied by a significant increase in the level of ROS. These alterations further contribute to the dysregulation of mitochondrial dynamics. Notably, Drp-1 has been implicated in the development of cardiac hypertrophy through excessive production of ROS. Conversely, reducing the expression of mitochondrial function-related proteins Fis-1 and Drp-1 may exert a cardioprotective effect [31,32]. The destruction of mitochondrial structure and dysregulation of function is closely related to the elevation of oxidative stress [33].

NADPH oxidase (NOX), a pivotal enzyme involved in redox signaling, is selectively expressed by mitochondria and facilitates the conversion of oxygen molecules into ROS [34]. 4-HNE is one of the main end products of lipid peroxidation, and its content reflects the degree of tissue oxidative damage [35]. Nitric oxide (NO) is a soluble and highly diffused gas, which is a key regulator of cardiac function. The excessive production of i-NOS, an enzyme responsible for NO synthesis, could lead to cytotoxicity and chronic negative inotropic effects on cardiomyocytes [36,37]. Keap1 functions as a constituent of E3 ubiquitinase and, under normal circumstances, it interacts with the target gene Nrf2 to facilitate ubiquitination and subsequent degradation by the proteasome. However, when an imbalance between oxidation and antioxidants occurs, the cysteine residues of Keap1 undergo modifications that alter its conformation. This conformational change leads to the release of Nrf2 into the nucleus, thereby inducing the expression of downstream antioxidant enzymes, such as HO-1 [38,39]. The findings from our study indicated that in mice subjected to CIH, there was a significant increase in the levels of 4-HNE and i-NOS, suggesting the presence of oxidative stress and lipid peroxidation. However, the administration of H_2_ effectively mitigated the development of oxidative stress by activating the Keap1-Nrf2-HO-1 pathway, ultimately leading to an improvement in myocardial damage (Figure 7).

Iron is an essential transition metal that fulfils a significant function in upholding optimal mitochondrial function through its involvement in energy metabolism [40]. However, an excessive buildup of iron beyond the capacity of iron storage results in the generation of substantial LIP (specifically free Fe^2+^), which intensifies the production of ROS and oxidative damage [41]. Excessive iron has been demonstrated to have a direct impact on excitation–contraction coupling in cardiomyocytes, which might account for diastolic dysfunction [40]. Additionally, the production of ROS through iron catalysis could elevate levels of LIP by binding iron into Fe-S clusters or other responsive forms [42,43]. Cellular iron homeostasis is mainly achieved by controlling the uptake by TfR1 and DMT1 and the export by FPN1 [44]. Our findings indicated that CIH led to an upregulation in the expression of TfR1, FTL, and MtFt proteins, while downregulating the expression of FPN1. Furthermore, CIH significantly increases iron deposition, which could be mitigated by H_2_ treatment, leading to a reversal in the expression of these proteins (Figure 7).

Previous research has demonstrated that H_2_ could hinder iron overload in renal tubular epithelial cells and mitigate renal injury by modulating the expression of HIF-1α, hepcidin, and FPN1 proteins [12]. Other investigations indicated that chronic heart failure patients exhibited abnormally elevated serum hepcidin levels, and the alterations might lead to anemia, consequently leading to diminished cardiac function and unfavorable prognosis [45]. Hepcidin serves as a crucial regulatory hormone regulating the body’s iron status. Elevated levels of hepcidin can impede the expression of FPN1 protein, resulting in a reduction in iron release and exacerbating iron deposition [46]. Our previous study demonstrated that CIH exposure caused an increase in hepcidin levels, accompanied by an increase in iron accumulation in hippocampus tissue [47]. In this study, following treatment with H_2_, we observed a decline in hepcidin levels, as well as an upregulation of FPN1 protein, which effectively controlled iron deposition (Figure 7).

The study demonstrated that the mice with cardiomyocyte-specific deletion of the FPN1 gene could develop left ventricular dysfunction at three months old. When the TfR1 was downregulated, it was not enough to alleviate the iron overload caused by FPN1 deficiency. This suggested that iron release is an essential component of cardiomyocyte iron metabolism [40,48]. The regulation of FPN1 is also influenced by the level of transcription. The 5′-untranslated region (UTR) of *FPN1* mRNA contains an iron-responsive element (IRE), which allows for the regulation of FPN1 expression at the post-transcriptional level through the interaction between IRPs and the IRE system. An increase in cellular iron content leads to a decrease in IRP-2 expression, an upregulation of FPN1 expression, a reduction in iron absorption by cells, an increase in iron output, an augmentation of iron storage, and mitigation of iron deposition [49,50]. FBXL5 is a significant enzyme involved in the regulation of ubiquitination and degradation of IRP-2. Following an elevation in oxidative stress levels, the upregulation of FBXL5 could induce the ubiquitination and degradation of IRP-2 protein expression, thereby enhancing the cellular capacity for iron storage. Consequently, although the capacity for iron intake is diminished, the augmented iron storage capacity results in the accumulation of intracellular iron [51]. DMT1 is also known to be involved in the absorption of iron in small intestinal epithelial cells and the translocation of iron from endocytosis to the cytosol [52]. The *DMT1* mRNA exists in two forms, namely *DMT1(+ire)* and *DMT1(-ire)*. The DMT1(+ire) form primarily functions in cellular iron uptake and intracellular iron transport. Research has demonstrated that the hypoxia response element (HRE) located in exon 1B of the DMT1 promoter region was targeted by HIF-1α, and the regulation of DMT1 expression by hypoxia could impact cellular iron intake [53]. Our studies showed that activated FBXL5 and degraded IRP-2 protein levels, increased HIF-1protein and *DMT1(+ire)* mRNA in cardiac tissue exposed to CIH, further causing iron deposition; nevertheless, it has all been reversed by H_2_ treatment. In addition, a study demonstrated iron overload was a common denominator in patients of heart failure. When the iron is overloaded, the circulating non-transferrin-bound iron (NTBI) levels are high, and Fe^2+^ is taken up into cardiomyocytes through L-type calcium channels (LTCCs), a route of uptake that is not regulated by IRPs [40].

However, this article still exhibits certain limitations. Firstly, quantifying the extent of H_2_ absorption within the animal body or cells proves challenging, necessitating reliance on indirect indicators for evaluating the protective effects of H_2_. Secondly, due to hydrogen’s low molecular weight and facile diffusion, discerning its interaction and mode of action with specific molecules poses a formidable task. Consequently, future investigations will prioritize the exploration of hydrogen’s mode of action.

## 5. Conclusions

In conclusion, our experiment indicated a potential correlation between elevated levels of iron within cardiomyocytes and the occurrence of oxidative stress and impaired mitochondrial function following exposure to CIH. Hydrogen has been observed to mitigate cellular iron accumulation by upregulating the expression of FPN1, thereby alleviating cardiac hypertrophy and mitochondrial impairment induced by CIH. These discoveries offer additional justification for the therapeutic intervention of cardiovascular damage in patients with OSA.

## Figures and Tables

**Table 1:** The Sequence of Primers Used for the Expression of Genes.

Gene	Forward	Reverse	Length
*β-actin*	AGGCCCAGAGCAAGAGAGGTA	TCTCCATGTCGTCCCAGTTG	81 bp
*Nppa*	GGGTAGGATTGACAGGATTGG	CCTCCTTGGCTGTTATCTTC	79 bp
*Nppb*	ATCCGTCAGTCGTTTGGG	CAGAGTCAGAAACTGGAGTC	84 bp
*MYH7*	TGTTTCCTTACTTGCTACCC	GGATTCTCAAACGTGTCTAGTG	115 bp
*Fis-1*	AATATGCCTGGTGCCTGGTT	GCTGTTCCTCTTTGCTCCCT	102 bp
*Drp-1*	AGGTTGCCCGTGACAAATGA	TCAGCAAAGTCGGGGTGTTT	86 bp
*Opa-1*	GTGACTATAAGTGGATTGTGCCTG	AACTGGCAAGGTCTTCTGAGC	105 bp
*FPN1*	TGGATGGGTCCTTACTGTCTGCTA	TGCTAATCTGCTCCTGTTTTCTCC	139 bp
*DMT1(+ire)*	ACAGCCCAGGAGACCTTAAGAACA	ACCTTTGAACAAGCTCACCTCCGA	97 bp
*DMT1(−ire)*	CGCCCAGATTTTACACAGTG	TTGGAGTGTCGGTGCTTAAA	91 bp
*hepcidin*	AGACATTGCGATACCAATGCA	GCAACAGATACCACACTGGGAA	108 bp

## Data Availability

The data utilized to substantiate the study’s conclusions are provided in the article.
